# EZH2 alteration driven by microRNA-524-5p and microRNA-324-5p promotes cell proliferation and temozolomide resistance in glioma

**DOI:** 10.18632/oncotarget.21996

**Published:** 2017-10-24

**Authors:** Tongle Zhi, Tianfu Yu, Minhong Pan, Er Nie, Weining Wu, Xiefeng Wang, Ning Liu, Yongping You, Yingyi Wang, Junxia Zhang

**Affiliations:** ^1^ Department of Neurosurgery, The First Affiliated Hospital of Nanjing Medical University, Nanjing, China; ^2^ Department of Pathology, The First Affiliated Hospital of Nanjing Medical University, Nanjing, China

**Keywords:** EZH2, miRNA, prognosis, TMZ, glioma

## Abstract

Recent data have been shown that EZH2 is a critical oncogene via the repression of tumor suppressor genes in human cancers. In our study, we performed a genome-wide miRNA screen with a bioinformatics analysis to identify EZH2 specific miRNAs. Of these miRNAs, miR-524-5p and miR-324-5p were decreased in glioma tissues, and confered poor prognosis for glioma patients. Upregulation of miR-524-5p and miR-324-5p reduced glioma cell proliferation and increased temozolomide (TMZ) chemosensitivity by targeting EZH2. Importantly, the effection of miR-524-5p and miR-324-5p on cell proliferation and TMZ chemosensitivity in glioma were reversed by expression of EZH2 cDNA. Further, miR-524-5p and miR-324-5p overexpression suppressed glioma growth and prolonged survival in an intracranial xenograft model. Multivariate Cox regression analysis revealed that miR-524-5p was an independent prognostic factor in gliobalstoma patients. Taken together, these data indicate that miRNA-driven EZH2 repression may provide evidence of the molecular mechanism for gliomagenesis and the novel therapeutic targets for glioma.

## INTRODUCTION

The methyltransferase enhancer of zeste homolog 2 (EZH2), is the core catalytic element of polycomb repressive complex 2 (PRC2), and plays an important role in the regulation of cancer initiation, progression, invasion, and drug resistance. EZH2 catalyzes histone H3 lysine 27 methylation to form H3K27me3, and act as an oncogene via the repression of tumor suppressor genes in human cancers [1–3]. EZH2 overexpression has been shown in colorectal cancers [4], esophageal cancers [5], bladder cancers [6], and lung cancers [7]. Associations have also been reported between EZH2 overexpression and poor prognosis in esophageal cancers [8], breast cancers [9], renal cell carcinomas [10] and childhood intracranial ependymoma [11]. Our recent data have shown that increased EZH2 expression was associated with glioma grade, and high expression of EZH2 in GBM (glioblastoma) was determined to be a strong and independent predictor of short overall survival [12].

MicroRNAs constitute a class of small non-coding RNA molecules that function as post-transcriptional gene regulators and have been increasingly recognized as oncogenes or tumor suppressors [13, 14]. In human colon cancer, miR-506 inhibits cell proliferation and metastasis by binding the 3′UTR of EZH2 [15]. And in liver cancer, miR-101 represses tumor progression and sensitizes cancer cells to chemotherapeutic treatment through directly targeting EZH2 [16]. Also miR-101 is down-regulated in GBM cells, resulting in increased EZH2 expression and enhanced GBM cell proliferation, migration, and angiogenesis [17]. However, few studies of systematic mining EZH2 specific miRNA signature in human cancers have been reported. Thus, in this manuscript, we identified EZH2 specific miRNAs using integrated analysis of miRNA and mRNA arrays. Of these miRNAs, miR-524-5p and miR-324-5p confered poor prognosis for glioma patients and inhibited glioma cell proliferation by targeting EZH2.

## RESULTS

### MiRNA expression profiling for EZH2 specific miRNA signature

**Figure 1:**
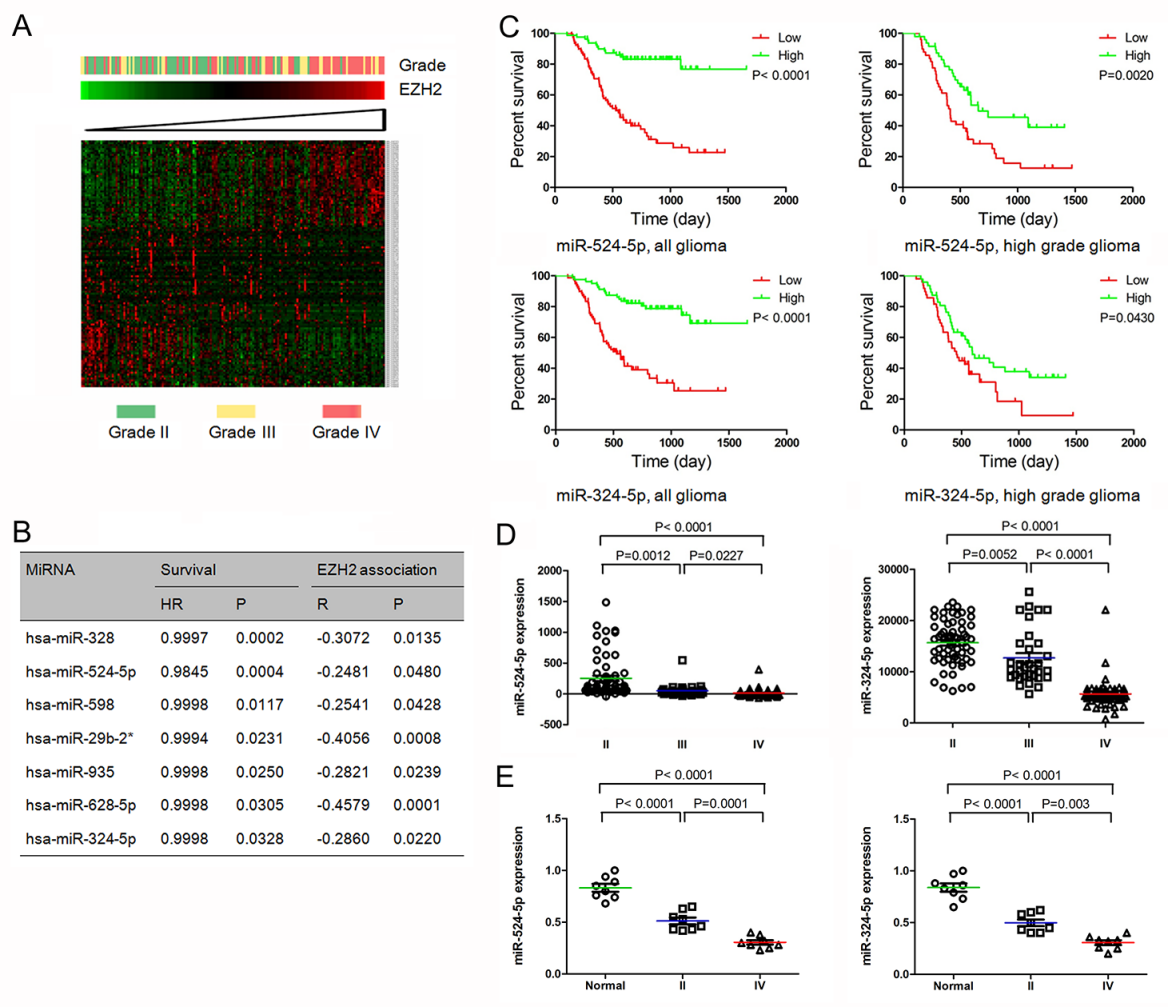
MiR-524-5p and miR-324-5p were identified as key EZH2 specific miRNAs **(A)** Heatmap of EZH2 and EZH2 associated miRNAs in glioma patients sorted by level of EZH2 expression in CGGA data. **(B)** Seven EZH2 specific miRNAs confer a better prognosis in GBM patients. **(C)** Kaplan-Meier survival curves for miR-524-5p and miR-324-5p in all gliomas and high grade gliomas. Patients with low levels of miR-524-5p or miR-324-5p had a significantly worse outcome. **(D)** Levels of miR-524-5p and miR-324-5p were analyzed in different glioma tissues in CGGA data. **(E)** Levels of miR-524-5p and miR-324-5p were analyzed in normal brain tissues and glioma tissues by PCR.

To identify the signature of EZH2 associated miRNA, miRNA and mRNA expression profiling of 64 GBM specimens from CGGA was employed. Pearson correlation was performed to analyze the relationships of EZH2 and all miRNA values using matlab software. 57 positively and 71 negatively correlated miRNAs with EZH2 expression were detected. Then heat map of these miRNAs was shown in profiling data with 158 gliomas (Figure 1A). Only the miRNAs with negatively significant correlation with EZH2 level were considered as EZH2 specific miRNA signature.

We analyzed the overall survival of GBM patients with EZH2 specific miRNA signature using matlab software, and found 7 miRNAs overexpression confer a better prognosis in GBM patients, as shown in Figure 1B. Further we used miRNA target analysis to explore whether EZH2 was a direct target of these miRNAs. Among them, two miRNAs (miR-524-5p and miR-324-5p), were predicted to bind to EZH2 3’UTR. The samples expressing lower level of miR-524-5p were associated with decreased survival relative to those with higher level in all gliomas (P < 0.0001) and high grade gliomas (grade III and grade IV) (P = 0.0020) (Figure 1C). And similar results of miR-324-5p were detected in all gliomas (P < 0.0001) and high grade glioma (P = 0.0430) (Figure 1C). These data indicate that miR-524-5p and miR-324-5p overexpression correlates with a significantly better survival outcome.

Further we check miR-524-5p and miR-324-5p expression in 158 glioma tissues. The level of miR-524-5p was significant lower in high grade gliomas than in low grade gliomas (P < 0.0001) (Figure 1D). Moreover, GBM demonstrated a significant decrease in miR-524-5p expression compared with that observed in low grade gliomas (P < 0.0001) and anaplastic gliomas (grade III) (P = 0.0227). And miR-324-5p was also inversely associated with tumor grade (P < 0.0001). Also, we checked 8 normal brain tissues, 8 grade II glioma tissues and 8 grade IV glioma tissues, and found the similar data shown in Figure 1E. Over all, these findings suggest that EZH2 specific miRNAs miR-524-5p and miR-324-5p may play an important role in glioma development.

### Critical role of miR-524-5p and miR-324-5p in glioma cell proliferation

**Figure 2:**
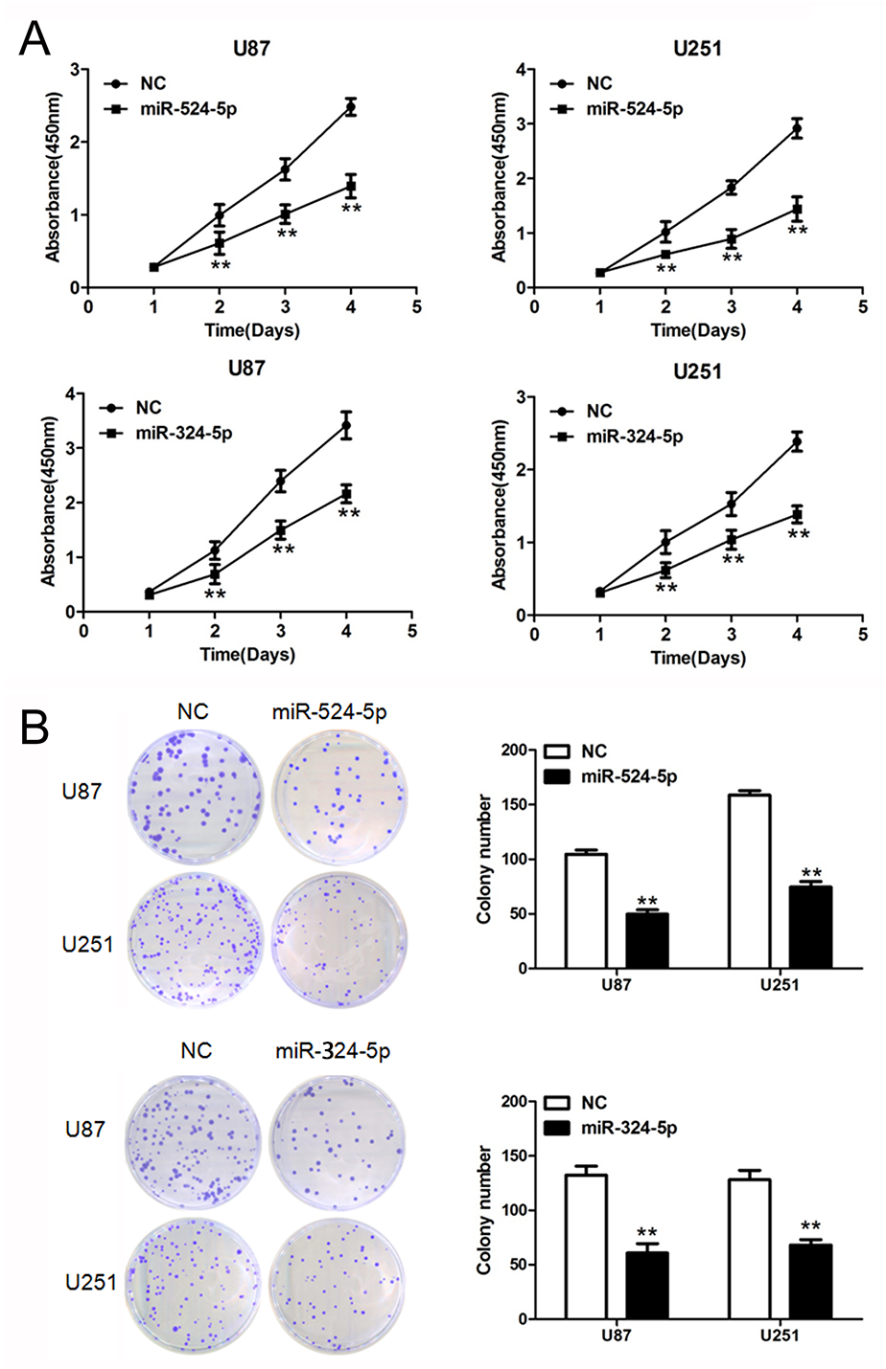
Overexpression of miR-524-5p or miR-324-5p inhibits tumor growth *in vitro* **(A)** Cells were treated with miR-524-5p or miR-324-5p mimics, and subjected to MTT assay. Data are presented as the means of triplicate experiments. **(B)** Cells were treated with miR-524-5p or miR-324-5p mimics, and subjected to colony formation assay. Data are presented as the means of triplicate experiments, ^**^ P <0.01.

To further explore the role of miR-524-5p and miR-324-5p in cell proliferation, we performed MTT and colony formation assays. Overexpression of miR-524-5p significantly inhibited glioma cell proliferation in U87 and U251 cells by MTT assay (Figure 2A). And cells exhibited a significant reduction in colony formation after 2 weeks of miR-524-5p treatment compared with the control group (Figure 2B). Also MTT and colony formation assays showed that miR-324-5p overexpression suppresses cell proliferation in both U87 and U251 glioma cells (Figure 2A and 2B).

### EZH2 is a direct target of miR-524-5p and miR-324-5p

**Figure 3:**
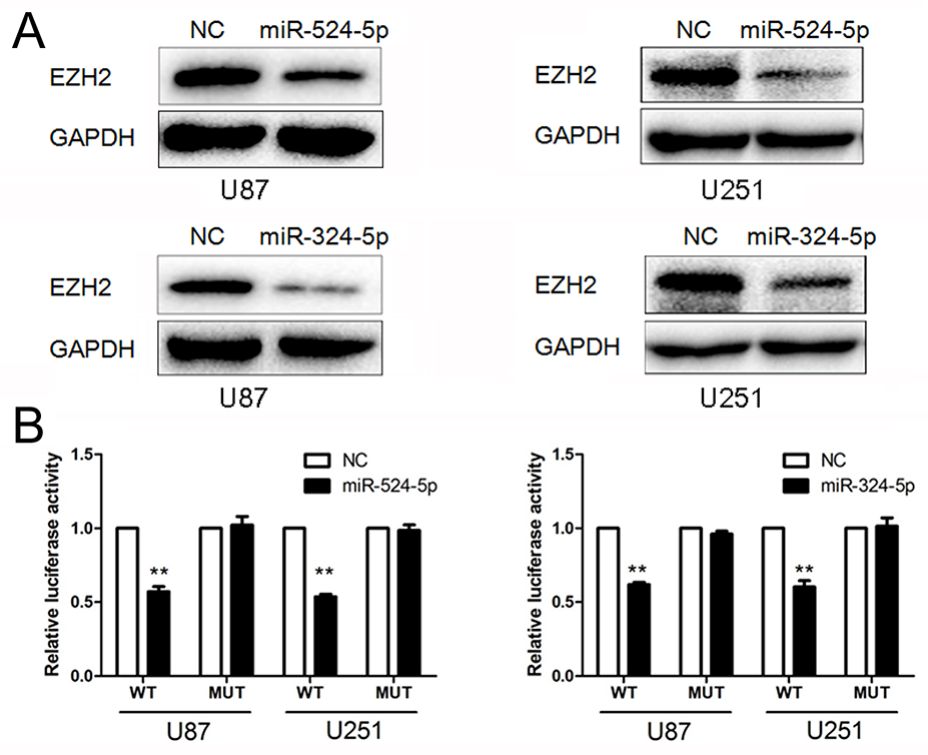
MiR-524-5p and miR-324-5p target EZH2 **(A)** Western blot analysis of lysates from cells transfected by miR-524-5p or miR-324-5p mimics probed with EZH2 antibody. GAPDH was served as the loading control. **(B)** pGL3-WT-EZH2-3′UTR-Luc and pGL3-MUT-EZH2-3′UTR-Luc reporters were transfected into glioma cells treated by miR-524-5p or miR-324-5p mimics. Luciferase activity was determined 48 h after transfection. The ratio of normalized sensor to control luciferase activity was shown. Error bars represent standard deviation and were obtained from three independent experiments, ^**^ P <0.01.

To determine the association of miR-524-5p and miR-324-5p with EZH2, Western blot and reporter assays were used. Western blot analysis showed that EZH2 expression was down-regulated in glioma cells over-expressing miR-524-5p or miR-324-5p (Figure 3A). In addition, we created pGL3-WT-EZH2-3′UTR and pGL3-MUT-EZH2-3′UTR plasmids for miR-524-5p or miR-324-5p, respectively. Reporter assays revealed that induction of miR-524-5p or miR-324-5p triggered a marked decrease of luciferase activity from pGL3-WT-EZH2-3′UTR but produced no change in the luciferase activity from pGL3-MUT-EZH2-3′UTR (Figure 3B). These data indicate that miR-524-5p or miR-324-5p directly modulated EZH2 expression by binding to the respective 3′UTR.

### Expression of EZH2 overrides miR-524-5p and miR-324-5p function

**Figure 4:**
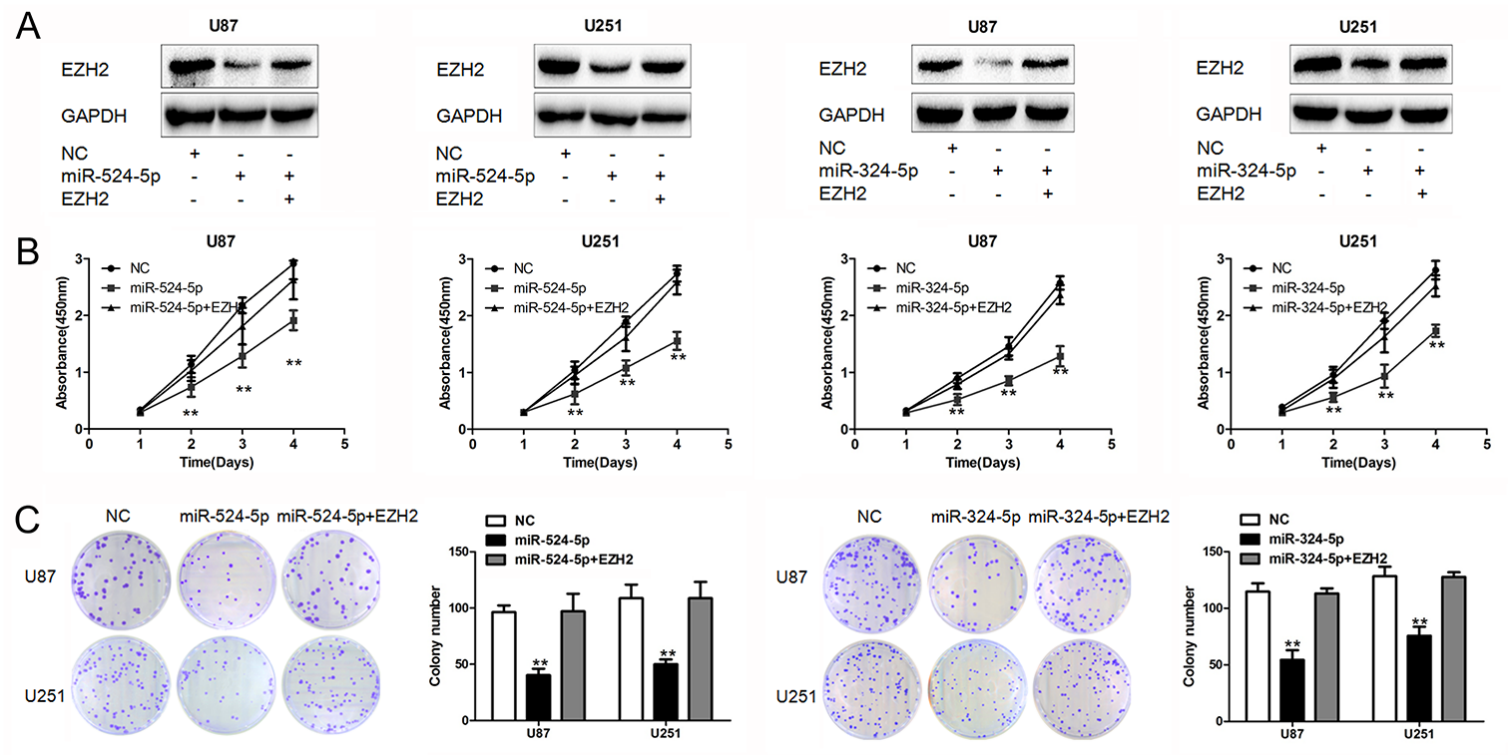
EZH2 is crucial for miR-524-5p and miR-324-5p signaling in glioma **(A)** Western blot analysis of lysates from cells transfected by miR-524-5p or miR-324-5p mimics alone, or in combination with EZH2 cDNA probed with EZH2 antibody. GAPDH was served as the loading control. **(B** and **C)** MTT and colony formation assays were measured in U87 and U251 glioma cell lines after the cells were treated with miR-524-5p or miR-324-5p mimics alone, or in combination with EZH2 cDNA. Results are representative of at least three independent experiments, ^**^ P <0.01.

Having demonstrated the profound effects of miR-524-5p and miR-324-5p on tumor suppression, we sought to examine the importance of EZH2 in miR-524-5p and miR-324-5p mediated cell proliferation. We transfected miR-524-5p mimics or miR-324-5p mimics together with and without EZH2 plasmid lacking 3′UTR into glioma cells (Figure 4A). Overexpression of EZH2 significantly rescued cell proliferation in U87 and U251 cells (Figure 4B and 4C). Similarly, expression of EZH2 largely abrogated miR-324-5p effects on cell proliferation (Figure 4B and 4C). These suggest that EZH2 is a critical target of miR-524-5p and miR-324-5p involved in glioma cell proliferation.

### MiR-524-5p and miR-324-5p increase TMZ chemosensitivity in glioma

**Figure 5:**
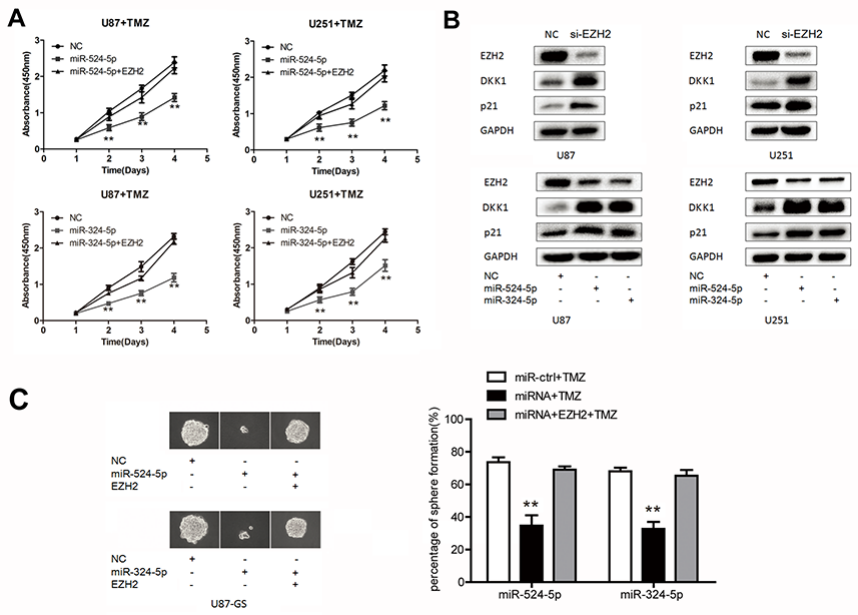
MiR-524-5p and miR-324-5p increases TMZ chemosensitivity in glioma **(A)** MTT assay was measured in U87 and U251 glioma cell lines by TMZ treatment after the cells were transfected with miR-524-5p or miR-324-5p mimics alone, or in combination with EZH2 cDNA. **(B)** Western blot analysis of lysates from cells transfected by si-EZH2, miR-524-5p or miR-324-5p mimics probed with EZH2, DKK1 and p21 antibody. GAPDH was served as the loading control. **(C)** Clonogenic assay was used to measure the sphere formation of glioma stem-like cells treated TMZ for 15 d. Representative photomicrographs of new clonal sphere (left) and statistical analysis of the percentage of positive wells (right). Results are representative of at least three independent experiments, ^**^ P <0.01.

Recent data have shown that inhibition of EZH2 promotes chemotherapeutic drug temozolomide (TMZ) chemosensitivity in glioblastoma cells [18]. Thus, we explore whether miR-524-5p and miR-324-5p play key roles in TMZ resistance. The MTT assay showed that upregulation of miR-524-5p significantly reduced the viability of U87 and U251 cells after TMZ treatment (Figure 5A). And EZH2 overexpression could reverse miR-524-5p role in TMZ chemosensitivity. Also, the same results of miR-324-5p in TMZ chemosensitivity were observed (Figure 5A). Further, we evaluated the expression levels of DKK1 and p21, which were targets of EZH2 [19, 20] and associated with chemosensitivity of alkylating drugs [21, 22]. EZH2 reduction elevated DKK1 and p21 were elevated when cells were treated by si-EZH2, miR-524-5p and miR-324-5p mimics, as shown in Figure 5B. Glioma stem-like cells has been identified as a main object for TMZ resistance. Thus, we performed clonogenic assay to detect the effect of TMZ on glioma stem-like cells (U87-GS cells). Compared to the control group, the percentage of positive wells (positive well means 1 cell colony/well) was lower, and the spheres were smaller in miR-524-5p or miR-324-5p treated U87-GS cells under TMZ treatment (Figure 5C). EZH2 overexpression could eliminate miR-324-5p or miR-324-5p effects on sphere formation under TMZ treatment. These suggested that miR-524-5p and miR-324-5p may modulate TMZ resistance in glioma cells.

### MiR-524-5p and miR-324-5p suppress tumor growth in an intracranial xenograft model

**Figure 6:**
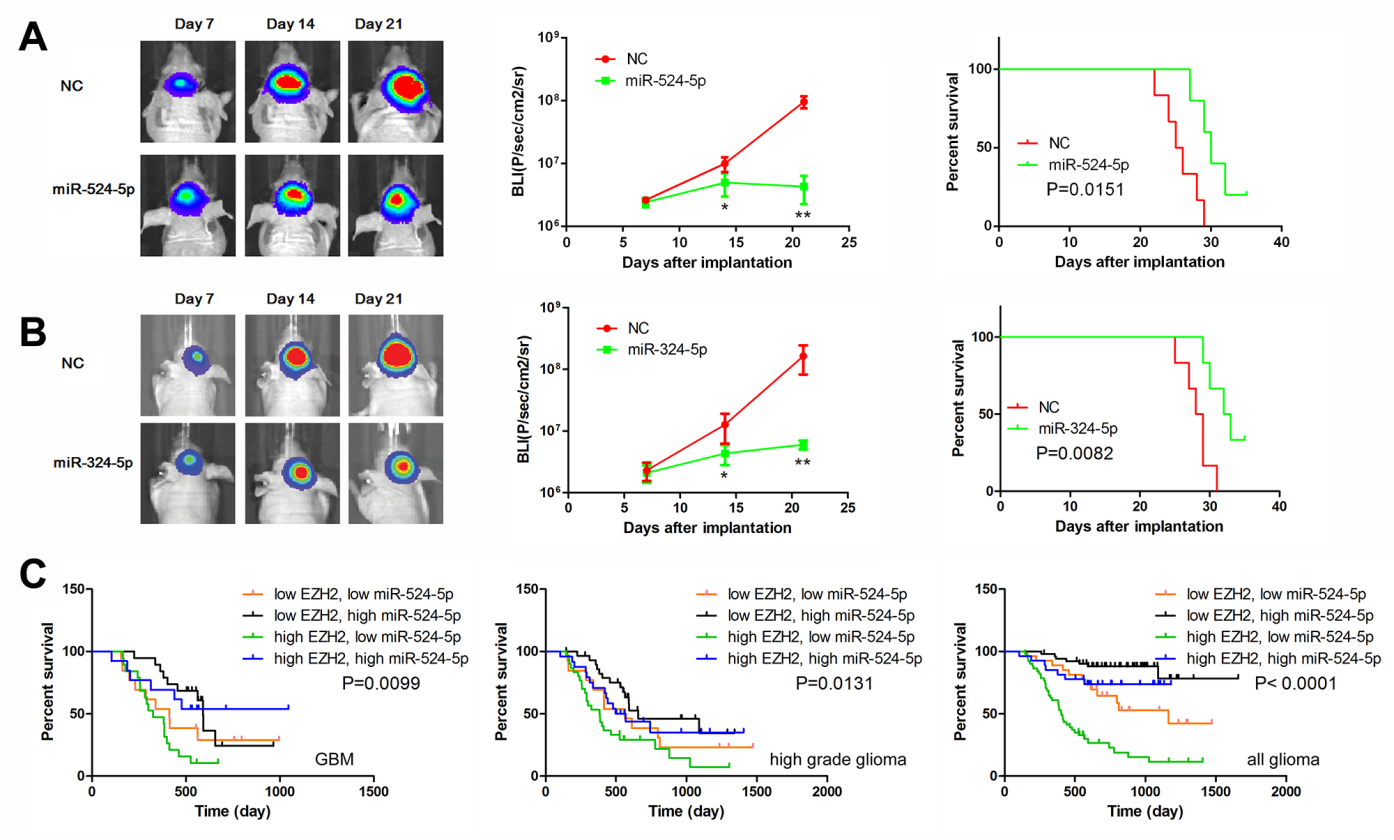
MiR-524-5p suppresses tumor growth in an intracranial xenograft model **(A, B)** U87 cells pretreated with lentivirus containing a luciferase reporter were implanted into the brains of nude mice, and tumor formation was assessed by bioluminescence imaging. Changes in bioluminescent signal were detected at days 7, 14, and 21 after implantation. Overall survival of nude mice was determined by Kaplan–Meier survival curves and log-rank test was used to assess the statistical significance of the differences. ^*^ P <0.05, ^**^ P <0.01. **(C)** Combining analysis of miR-524-5p and EZH2 expression by Kaplan-Meier survival curves in different glioma patients of CGGA data.

To further evaluate the effects of miR-524-5p and miR-324-5p on tumor growth *in vivo*, we established intracranial xenograft tumors in nude mice. U87 cells were pretreated with a lentivirus containing a luciferase reporter. As shown in Figure 6A, when miR-524-5p was overexpressed, the intracranial tumor significantly decreased compared with the corresponding control group (P < 0.01). Compared with the control group, miR-524-5p-treated group showed prolonged survival until the end of the observation (on day 35) (Figure 6A). The similar results of miR-324-5p were observed (Figure 6B). Furthermore, immunohistochemistry assay showed the decreased expression of EZH2 and increased expression of p21 and DKK1 after overexpression of miR-324-5p or miR-524-5p, which were consistent with the *in vitro* results (Supplementary Figure 1). Taken together, these findings demonstrate that both miR-524-5p and miR-324-5p inhibited glioma malignant progression *in vivo*.

### MiR-524-5p is an independent prognostic factor in GBM patients

As shown in Supplementary Table 1, high expression of miR-524-5p was significantly associated with IDH1 mutation, PCNA expression, but not associated with MGMT promoter methylation. Then we performed univariate cox regression analysis using clinical and genetic variables for 64 GBM patients. We found that high expression of miR-524-5p, high KPS score and total resection were statistically associated with overall survival, while IDH1 mutation and MGMT promoter methylation were not associated with overall survival (Table 1). Multivariate Cox regression analysis revealed that miR-524-5p expression, Ki-67 expression and total resection were correlated independently with overall survival when considering gender, KPS score, IDH1 mutation, EGFR expression and PCNA expression (P < 0.3, univariate cox regression analysis).

**Table 1:** Cox regression analyses of miR-524-5p expression and pathologic characteristics in relation to overall survival in GBM

Variable	Univariable regression		Multivariable regression	
	HR	P	HR	P
Gender (female/ male)	1.440	0.276	1.542	0.287
Increasing age	1.008	0.541		
KPS score (>80)	0.363	0.001	0.496	0.089
Total resection	0.550	0.022	0.360	0.031
IDH1 mutation	0.494	0.142	0.667	0.479
MGMT promoter methylation	1.208	0.645		
High miR-524-5p	0.403	0.005	0.336	0.012
High Ki-67	1.059	0.050	2.529	0.027
High EGFR	1.483	0.223	0.871	0.732
High MGMT	1.077	0.823		
High MMP9	0.744	0.421		
High P53	1.165	0.688		
High PCNA	1.785	0.069	1.805	0.131

### MiR-524-5p could be used to sub-classify gliomas in combination with EZH2 expression

To further detail the role of miR-524-5p in glioma survival, we designed a glioma classification model based on miR-524-5p and EZH2 expression level. As shown in Figure 6C, GBM patients with low EZH2 and high miR-524-5p had the longest OS, whereas GBM patients with high EZH2 and low miR-524-5p had the shortest (median OS = 529.6 vs. 349.8 days; logrank test, P = 0.0099). And the similar results in high grade gliomas and all gliomas were observed. High EZH2 and low miR-524-5p in glioma patients was associated with the longest OS (median OS = 460.6 days; logrank test, P < 0.0001). Therefore, we conclude that classification combining miR-524-5p and EZH2 expression represents a more precise biological property and prognosis.

## DISCUSSION

Here, we present the first study to integrate a genome-wide miRNA screen with a bioinformatics analysis derived from clinical specimens to identify clinically pertinent EZH2 specific miRNAs. To our surprise, 71 miRNAs are negatively significant correlated with EZH2 level and 7 EZH2 specific miRNAs confer a better prognosis in GBM patients. We characterize two candidates, miR-524-5p and miR-324-5p, which bind to EZH2 3’UTR to down-regulate EZH2 expression, thereby suppressing glioma cell proliferation. Importantly, restoring EZH2 expression could override miR-524-5p and miR-324-5p function in glioma cells. These data indicate that miRNA-driven EZH2 repression may underlie the molecular mechanism for gliomagenesis and represent a novel therapeutic target for glioma.

Accumulating data have been validated that miR-524-5p is a key tumor suppressors in several human cancers. In melanoma cells, miR-524-5p directly interacts with the 3’-untranslated regions of both BRAF and ERK2 to inhibit MAPK/ERK signaling, cell proliferation, and cell migration [23]. Another study has shown that miR-524-5p behaves as a tumor suppressor by negatively targeting Jagged-1 and Hes-1 in gliomas [24]. Here, we found miR-524-5p is down-regulated in glioma tissues and associated with tumor grade. Low expression of miR-524-5p in GBM is determined to be a strong and independent predictor of short overall survival. Overexpression of miR-524-5p inhibits glioma cell growth *in vitro* and *in vivo*. Further a combined index of miR-524-5p and EZH2 expressions better reflects prognosis of glioma patients.

EZH2, as the core element of PRC2, mediates histone methylation and recruits DNA methyltransferase in the silencing of a variety of genes, including miRNAs [25, 26]. In gastric cancer and glioma cells, knockdown of EZH2 not only impacted H3K27 trimethylation but also reduced DNMT1 presence on the miR-200b/a/429 promoter [27]. In human nasopharyngeal carcinoma, EZH2 inhibits miR-1 transcription via promoter binding activity, leading to enhanced expression of Endothelin-1 (ET-1) which is suppressed by miR-1 targeting of ET-1 3’UTR [28]. In aggressive B-Cell lymphomas, Myc, HDAC3, and PRC2 are tethered to the miR-29 promoter regions as a co-repressor complex to down-regulate miR-29 expression through histone deacetylation and trimethylation [29]. In our results, miR-1, miR-29b, miR-29c are listed in EZH2 specific miRNAs. Thus, the detailed mechanism involved in EZH2 specific miRNAs in glioma progression needs further investigation.

In summary, we identify EZH2 specific miRNAs through a genome-wide miRNA screen. MiR-524-5p and miR-324-5p exhibit a strong tumor-suppressive effect by targeting EZH2. Further, miR-524-5p was an independent prognostic factor in GBM patients. These data indicate that miRNA-driven EZH2 repression provides evidence of the molecular mechanism for gliomagenesis and the novel therapeutic targets for glioma.

## MATERIALS AND METHODS

### Patients and samples

We performed miRNA and mRNA profiling upon 158 glioma samples collected from the Chinese Glioma Genome Atlas (CGGA, http://www.cgcg.org.cn/) including 61 gliomas of grade II, 33 gliomas of grade III, and 80 GBMs. 8 normal brain tissues, 8 grade II glioma tissues and 8 grade IV glioma tissues were obtained from The First Affiliated Hospital of Nanjing Medical University. This study was approved by the institutional review boards of all hospitals involved in the study, and written informed consent was obtained from all the patients who were selected.

### Cell culture and treatment

Human glioma cells (U87 and U251) were obtained from the Chinese Academia Sinica cell repository (Shanghai, China). Cells were maintained in Dulbecco's modified Eagle's medium (DMEM, Gibco) supplemented with 10% fetal bovine serum, and incubated at 37°C with 5% CO2. Glioma stem-like cells (U87-GS), single-cell populations which came from adherent U87 cells, were resuspended in DMEM/F12 (Gibco), supplemented with N2(1/100, Invitrogen), B27(1/50, Invitrogen), 10 ng/ml recombinant human basic fibroblast growth factor (FGF, Invitrogen) and 10 ng/ml recombinant human epidermal growth factor (EGF, Invitrogen). Oligonucleotides were chemically synthesized and purified by high-performance liquid chromatography (GenePharma, Shanghai, China). Then miRNA mimics were transfected using Lipofectamine 2000 (Invitrogen). Lentivirus overexpressing miR-524-5p and ontaining a luciferase reporter were obtained from genechem (Shanghai, China).

### Colony formation assay and clonogenic assay

4000 glioma cells from each group were seeded into new dishes. The medium was replaced every other day for 8 days, and the cells were stained with crystal violet at the end of the time course prior to the capture of the representative images via camera. U87-GS spheres were dissociated with TrypLETM Express (Gibco) and resuspended in sphere medium. Disaggregated spheres after trasfection were seeded in 96-well plates at clonal density (1 cell per well) and cultured in sphere medium with TMZ treatment. To renew growth factors supply, fresh sphere medium was added every 5 d. 15 d later, we counted the percentage of spheres formation. The assays were repeated at least 3 times.

### Western blot analysis

Equal amounts of protein per lane were separated by 8% SDS-polyacrylamide gel and transferred to PVDF membrane. The membrane was blocked in 5% skim milk for 1 h and then incubated with a specific antibody for 2 h. The antibodies used in this study were: antibodies to EZH2, DKK1, p21 (Cell Signaling Technology, USA). The antibody against GAPDH (Santa Cruz, USA) was used as control. The specific protein was detected by using a SuperSignal protein detection kit (Pierce, USA). The band density of specific proteins was quantified after normalization with the density of GAPDH.

### Luciferase reporter assay

The human β-catenin 3’UTR were amplified and cloned into the XbaI site of the pGL3-control vector (Promega, USA), downstream of the luciferase gene, to generate the plasmids pGL3-WT-EZH2-3′UTR. pGL3-MUT-EZH2-3′UTR plasmids were generated from pGL3-WT-EZH2-3′UTR by deleting the binding site. For the luciferase reporter assay, cells were cultured in 96-well plates, transfected with the plasmids and miRNA mimics using Lipofectamine 2000. 48h after transfection, luciferase activity was measured using the Luciferase Assay System (Promega).

### Nude mouse glioma intracranial model

U87 cells were transfected with lentivirus overexpressing miR-524-5p or miR-324-5p and ontaining a luciferase reporter *in vitro* for 2 days. A total of 5 × 10^5^ U87 cells infected with virus were implanted stereotactically to establish intracranial gliomas using cranial guide screws. Mice were imaged for Fluc activity using bioluminescence imaging (BLI) on day 7, 14 and 21.

### Statistical analysis

Descriptive statistics, including mean and ±SE, along with independent sample t-tests, and the Pearson correlation were used to determine significant differences. Kaplan-Meier analysis was employed to assess the survival rate of patients. P<0.05 was considered a significant difference.

## SUPPLEMENTARY MATERIALS FIGURES AND TABLES



## Abbreviations

GBM: Glioblastoma multiforme
miRNAs: MicroRNAs
CGGA: Chinese Glioma Genome Atlas
TMZ: Temozolomide
PRC2: Polycomb repressive complex 2
ET-1: Endothelin-1
BLI: Bioluminescence imaging
