# Supplementary material for: EZH2 alteration driven by microRNA-524-5p and microRNA-324-5p promotes cell proliferation and temozolomide resistance in glioma
Source: Oncotarget. 2017 Oct 24;8(56):96239–48. doi: 10.18632/oncotarget.21996 (PMC5707096; doi:10.18632/oncotarget.21996)
Supplement: Supplementary file 1 [file oncotarget-08-96239-s001.pdf]

EZH2 alteration driven by microRNA-524-5p and microRNA-324-5p promotes cell proliferation and temozolomide resistance in glioma

SUPPLEMENTARY MATERIALS

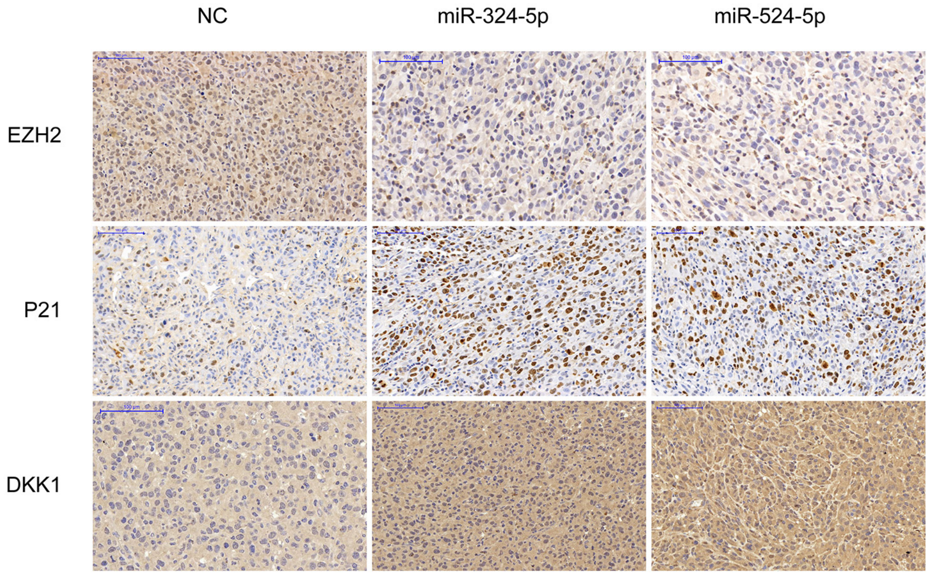

Supplementary Figure 1: Representative IHC images of EZH2, P21 and DKK1 in tissues from mice with orthotopic tumors derived from LV-miR-NC-U87, LV-miR-324-5p-U87 and LV-miR-524-5p-U87 cells. Magnification ×200.

Supplementary Table 1: Clinical and molecular pathology features of GBM samples in association with miR-524-5p expression

	Low	High	P value
No. of cases	32	32	
Gender (Female/ Male)	11/21	11/21	1
Age at diagnosis (Year)	44.6 ± 13.3	42.8 ± 12.8	0.250
KPS score (≥80/ <80)	20/12	23/9	0.424
MGMT promoter methylation (Unmethylated/ Methylated)	16/7	10/6	0.645
IDH1 mutation (Mutation/ No mutation)	2/29	8/15	0.012
Ki-67(Low/ High)	9/21	11/21	0.713
EGFR (Low/ High)	13/17	16/16	0.599
MGMT (Low/ High)	13/17	8/24	0.127
MMP9 (Low/ High)	6/24	7/25	0.856
P53 (Low/ High)	9/21	5/27	0.176
PCNA (Low/ High)	15/15	24/8	0.042
Overall Survival	390.9 ± 202.7	509.2±197.0	0.021
